# High sulfur content polymers: The effect of crosslinker structure on inverse vulcanization

**DOI:** 10.1002/pola.29067

**Published:** 2018-08-24

**Authors:** Jessica A. Smith, Xiaofeng Wu, Neil G. Berry, Tom Hasell

**Affiliations:** ^1^ Department of Chemistry University of Liverpool Crown Street Liverpool United Kingdom L69 7ZD

**Keywords:** crosslinker, Fukui indices, inverse vulcanization, polymers; radical reactions; sulfur–5‐ethylidene‐2‐norbornene copolymer, sulfur

## Abstract

The discovery of inverse vulcanization has allowed polymers to be made using elemental sulfur as the major component. However, until now, there has been little discussion of why seemingly similar crosslinkers result in polymers with radically different properties. Combining synthesis, spectroscopy, and modeling, this study reveals the structure–property relationships of sulfur polymers and reports a new system using 5‐ethylidene‐2‐norbornene as a crosslinker that can stabilize up to 90 wt % of elemental sulfur.

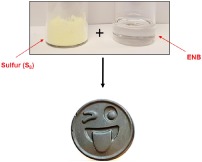

## INTRODUCTION

**Figure 1:**
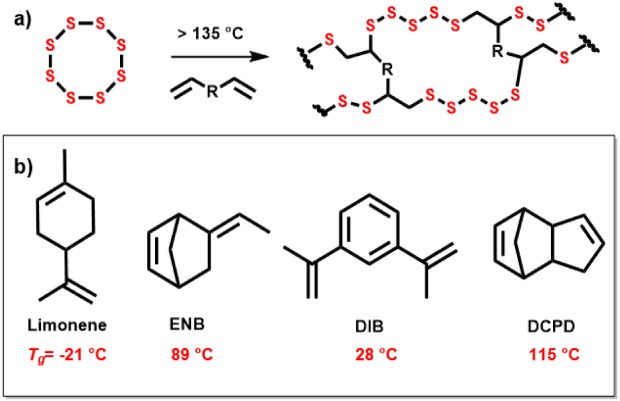
Inverse vulcanization reaction of elemental sulfur and crosslinker, where R indicates an organic molecule with unsaturated bonds (b) From left to right: limonene, ENB, DIB, and DCPD. *T*_g_s given below the crosslinkers are for 50 wt % to 50 wt % copolymers of the crosslinkers and sulfur. It can be noted that although the crosslinkers have similar molecular mass, and the same degree of unsaturation, they show markedly different properties. [Color figure can be viewed at http://wileyonlinelibrary.com]

Inverse vulcanized high sulfur content polymers have attracted much attention recently due to their potentially low cost and diverse applications. More than 60 million tonnes of excess sulfur are produced annually by hydrodesulfurization of crude oil and gas_._ 1 Conventional uses of sulfur only use a fraction of this supply. Production of polymeric materials from sulfur would alleviate this, but pure sulfur polymers are unstable and depolymerize back to S_8_.^1^ However, “inverse vulcanization,” first reported by Pyun and coworkers,^2^ allows the stabilization of sulfur polymers by a small organic molecule that acts as a crosslinker against depolymerization, such as 1,3‐diisopropenylbenzene (DIB) [Fig. 1(a,b)]. S‐DIB is a shape persistent and stable copolymer. However, DIB is relatively expensive in comparison with sulfur, and there has been interest to use lower cost crosslinkers, such as limonene [Fig. 1(b)].^3^ Limonene has many advantages; being bioderived, renewable, and economic. However, the sulfur–limonene polymer formed was a low molecular weight polysulfide, rather than a fully crosslinked high molecular weight polymer, and is not shape persistent [Supporting Information Fig. S1], which may limit some applications. Similarly, dicyclopentadiene (DCPD), an industrial by‐product, has been shown to produce stable polymers with sulfur, forming a hard brittle solid [Fig. 1(b)].^4^ It is also unclear why very structurally similar crosslinkers, in terms of molecular mass and degree of unsaturation, produce materials of dramatically different properties after reaction with sulfur, that is, from viscous liquids to rubbery or glassy solids.

Here, we present both computational and experimental data to investigate the structure–property relationships of a series of related crosslinkers in inverse vulcanization reactions. We compare crosslinkers: DCPD, DIB, and limonene [Fig. 1(b)], with a new structurally similar alternative 5‐ethylidene‐2‐norbornene (ENB) [Fig. 1(b)]. ENB was chosen as a potential crosslinker for comparison due to its structural similarities to DCPD (Fig. 1(b)], to gain a clearer insight into how inverse vulcanization may be controlled and occur. ENB is commonly used in the manufacturing of ethylene–propylene–diene terpolymers,^5^ and can be readily sourced in bulk. Potential applications of sulfur polymers are widespread, covering diverse areas such as heavy metal remediation,^3^ Li‐S batteries,^6^ lenses,^7^ thermal insulation,^8^ and self‐healing polymers.^9^

## RESULTS AND DISCUSSION

**Figure 2:**
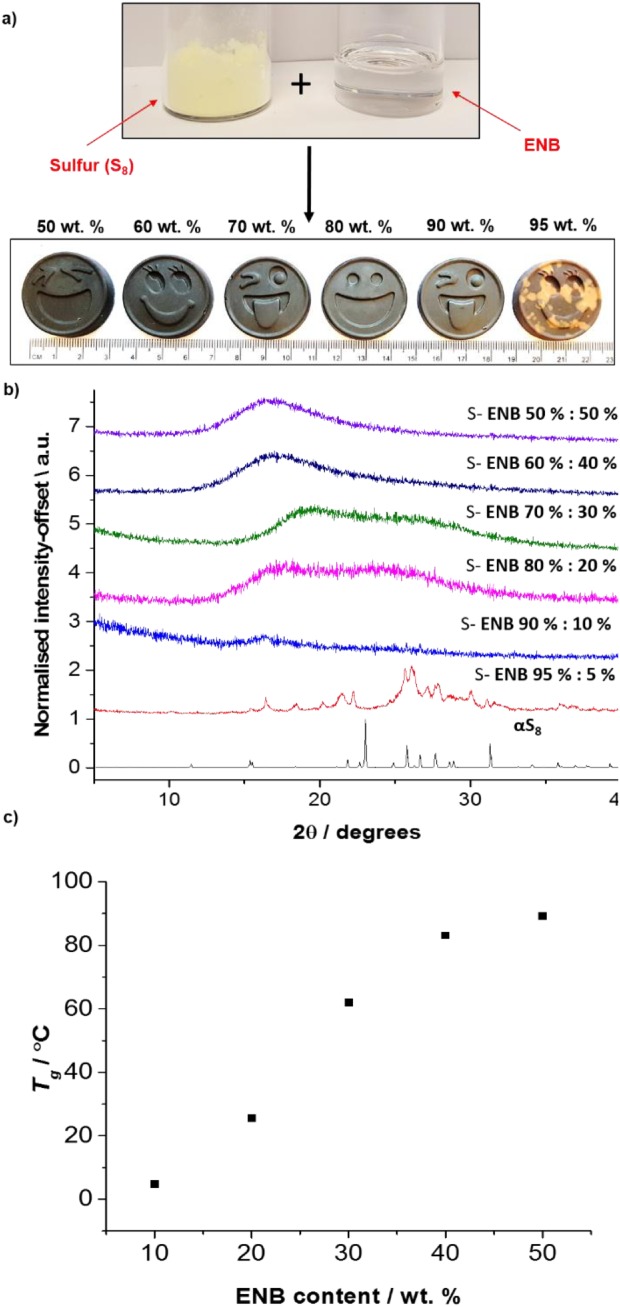
(a) Top: elemental sulfur (left) and ENB crosslinker (right). Bottom: S‐ENB polymers 50, 60, 70, 80, 90, and 95 wt % of elemental sulfur. (b) PXRD patterns of S‐ENB and elemental sulfur, showing amorphous materials up to 90 wt % S_8_. (c) The *T_g_* of S‐ENB polymers as function of ENB composition. [Color figure can be viewed at http://wileyonlinelibrary.com]

Inverse vulcanized S‐ENB copolymers were successfully prepared of ratios 90 wt %–50 wt % elemental sulfur [Fig. 2(a)]. This is a facile, efficient one pot synthesis that does not require any solvents or initiators to encourage polymerization. Powder X‐ray diffraction (PXRD) patterns [Fig. 2(b)] (see Supporting Information for details) for varying ratios of S‐ENB show no evidence of crystalline sulfur peaks, suggesting that the S‐ENB copolymers are stable against depolymerization of sulfur to S_8_, even at ratios of up to 90 wt % sulfur. This is a remarkable level of stabilization, in comparison with the majority of other inverse vulcanization crosslinkers reported recently,^10^ some of the best of which can stabilize only up to ∼80 wt % sulfur,^2^, ^4^ and many only 60 wt %,^11^ 50 wt %,^4^ and even 20 wt %.^11^ Of previously published high content sulfur polymers, it is only sulfur‐diallyl disulfide (SDA), that has proven to stabilize up to 90 wt % of elemental sulfur.^12^ S‐ENB provides a more readily sourced alternative to SDA, with comparable levels of sulfur stabilization. For samples in which sulfur bloom occurs (depolymerization of sulfur back to S_8_ crystals), we see diffraction peaks by PXRD, and a strong melting transition by differential scanning calorimetry [Supporting Information Figs. S2–S4). An S_8_ melting transition is clearly visible for the 95 wt % sulfur sample, but for the 90 wt % sulfur sample and below, no such pronounced peaks are seen. Elemental analysis (Supporting Information Table S2) corresponds well to the expected values, with a slight excess of sulfur likely caused by volatilization of crosslinker during reaction. Thermogravimetric analysis confirms complete reaction of sulfur, and a high char mass, increasing with crosslinker content, as would be expected for a crosslinked material (Supporting Information Fig. S5). FTIR (Supporting Information Fig. S6) shows either reduction or complete disappearance of the allylic =C—H and C=C stretching vibrations (∼3045 cm^−1^ and 1600 cm^−1^). This suggests successful polymerization between sulfur and ENB.

The glass transition temperatures (*T*_g_s) of S‐ENB copolymers were found to increase as a function of ENB composition [Fig. 2(c)], following a trend observed for other crosslinkers.^2^, ^3^, ^4^ The highest observed *T*_g_, for an equal mass of sulfur and ENB, was 89 °C—notably higher than that of S‐DIB (28 °C)^2^ and S‐limonene (–21 °C),^3^ but lower than S‐DCPD (115 °C)_._ 4 The relatively high *T*_g_ suggests S‐ENB may have more in common with S‐DCPD than S‐limonene and S‐DIB. It is not immediately apparent from the structures of the crosslinkers themselves why the resultant polymers would have such a range of *T*_g_s. However, a correlation has been noted in the degree of polymerization, crosslinking, and the *T*_g._ S‐limonene and S‐DIB show some solubility, with S‐limonene the lower in molecular weight of the two, while S‐DCPD becomes fully insoluble due to what is presumably a high molecular weight, more fully crosslinked structure.^4^ This is confirmed by solubility studies (Supporting Information Table S3), which show a complete lack of solubility in common organic solvents for S‐ENB. This poses the question, why do crosslinkers with similar molecular weights and the same number of available double bonds, result in such differences in behavior in the inverse vulcanization process?

The inverse vulcanization process has been said to be a bulk free radical copolymerization of unsaturated co‐monomers in liquid sulfur^6^; however, as with conventional vulcanization, the mechanism is complex and not yet fully understood. Both radical addition across the double bonds^2^, ^10^ and hydrogen abstraction have been proposed,^11^ and the nature of the reaction is likely to be temperature and crosslinker dependent.

**Figure 3:**
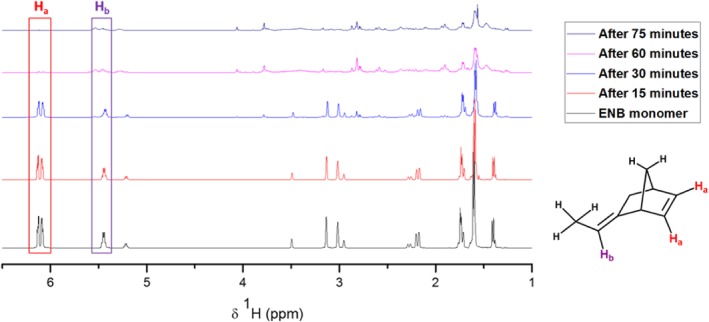
NMR kinetics experiment conducted at 135 °C. Approximately 20 mL aliquots were dissolved in CDCl_3_ and the soluble fraction was taken at 15, 30, 60, and 75 min. [Color figure can be viewed at http://wileyonlinelibrary.com]

**Figure 4:**
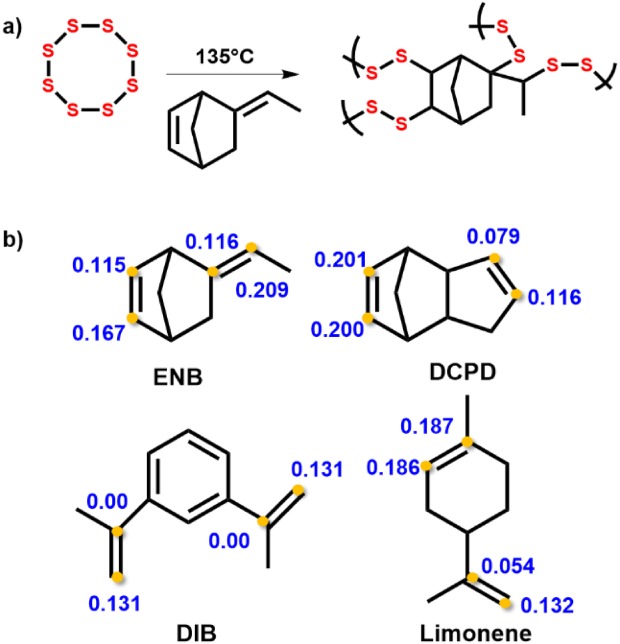
(a) Possible reaction pathway for S‐ENB. (b) Fukui indices of carbon atoms on each double bond on the following crosslinkers: limonene, ENB, DCPD, and DIB. [Color figure can be viewed at http://wileyonlinelibrary.com]

Sulfur‐olefin reactions are characterized as low temperature reactions up to about 140 °C, and high temperature reactions above 140 °C.^13^ It has been previously reported that reactions between sulfur and DCPD at 140 °C were found to produce soluble linear polymers, with the norbornene double bond being the most reactive at this temperature.^13^ Since then, it has also been found that in higher temperature reactions between sulfur and DCPD, an insoluble product is formed, suggesting reaction at both double bonds.^4^ NMR kinetics experiments were performed at different time intervals and temperature, to monitor the reaction of sulfur with ENB [Fig. 3] (see Supporting Information for details). Results at low temperatures show a decrease in magnitude of the H_a_ resonance (the norbornene double bond) suggesting that this double bond is indeed consumed preferentially at low temperature reactions, similarly to DCPD (see H_a_:H_b_ ratios in Supporting Information Tables S4 and S5). At a higher temperature, the reaction proceeds more rapidly (see Supporting Information, S7). In both reactions the H_b_ peak does not completely disappear, but shifts in position, indicative of a reaction taking place elsewhere on the molecule. Formation of peaks at ∼*δ* 4 ppm in both spectra [Fig. 3; Supporting Information Fig. S7) are indicative of S—C—H protons,^14^ further confirming the reaction between ENB and sulfur. However, these solution NMR results are for the soluble fraction only, and at an early reaction time. The insolubility of the material at longer reaction times suggests that reaction at the H_b_ position, though less favorable, does nevertheless occur [Fig. 4(a)].

The susceptibility of a double bond to reaction with sulfur radicals may determine the reaction temperature and rate, as well as the resultant molecular weight and degree of crosslinking. To further elucidate the reactivity of the crosslinkers, computational calculations were performed to predict sites of reactivity. Condensed Fukui indices were calculated to capture numerically the reactivity of each crosslinker [Fig. 4(b)]. The Fukui index predicts the reactivity of each atom in the molecule in a nucleophilic, electrophilic, or a radical sense (see Supporting Information for further details).^15^ Condensed Fukui values are calculated from atomic charges derived from electron density population analysis. Assessing how the calculated partial atomic charges change when an electron is added or removed to a system gives an indication of potential reactivity sites for radical attack.^15^ The larger the Fukui function the more susceptible the double bond is to radical attack. The computational calculations performed on the atoms in DCPD match what was expected from NMR kinetics experiments reported previously,^4^ with the norbornene double bond being more reactive than the cyclopentene. This difference in reactivity explains why low temperature reaction between sulfur and DCPD forms a linear polymer, where reaction has occurred only with the norbornene double bond and not the cyclopentene (Supporting Information Fig. S10).^13^ ENB, shows the carbon on the outer end of the exocyclic double bond to be more reactive than the other atoms in C=C double bonds. Despite this, the NMR kinetics experiment suggests that the norbornene double bond is consumed preferentially during the initial reaction. However, the Fukui indices [Fig. 4(b)] on all carbon atoms labeled do suggest both C=C are relatively reactive and susceptible to sulfur radical attack. This supports that S‐ENB crosslinks at both double bonds [Fig. 4(b)] rationalizing how it can stabilize a high proportion of sulfur and explaining the insolubility of the S‐ENB copolymers. Limonene and ENB both have similar molecular masses and possess two double bonds, but their resultant sulfur co‐polymers have considerably different chemical and physical properties. Chalker and coworkers depict reaction of sulfur with both exocyclic and endocyclic sites of limonene.^3^ This can be supported by the Fukui indices indicating each carbon atom on both double bonds show some susceptibility to sulfur radical attack [Fig. 4(b)]. If this was the case, a highly crosslinked, insoluble network would be expected. However, S‐limonene exhibits a low Tg (–21 °C), a high degree of solubility, and lack of shape persistency in comparison with similar sulfur‐polymers.^3^, ^4^, ^10^ A possible explanation for this could be limonene undergoing 1, 3‐hydrogen shifts and hydrogen loss to form an aromatic ring resulting in deactivation of the endocyclic site to sulfur crosslinking. Loss of one of the two reactive sites results in a more linear polymer, explaining the depression of the *T*_g_ and relatively high solubility (Supporting Information Figs. S11–S13).^16^ To test this, the gas emitted during the reaction between sulfur with limonene, DCPD, and ENB was collected (see Supporting Information). Production of H_2_S during inverse vulcanization was previously reported by Yagci and coworkers for the reaction of sulfur with polybenzoxazines.^11^ The reaction between limonene and sulfur produced a larger volume of gas than the other monomers, and triggered a connected H_2_S detector (Supporting Information Tables S6 and S7). This loss of hydrogen from limonene explains the aromatic signals observed in its NMR^3^ and is consistent with a more soluble, linear structure. Minimizing the production of poisonous H_2_S would be preferable in terms of industrial scale up and use, as previously discussed by Pyun and coworkers.^17^ In contrast, the structures of ENB and DCPD preclude such a hydrogen rearrangement and seem more stable against hydrogen abstraction by the sulfur, with both heteronuclear single quantum coherence (HSQC) and ^1^H NMR (Supporting Information Figs. S14–S16) confirming the absence of aromatic by‐products. The loss of hydrogen as H_2_S seems related to the reaction temperature required, with both following the trend S‐limonene > S‐DCPD > S‐ENB. The highest Fukui index for each crosslinker follows the opposite trend: S‐ENB > S‐DCPD > S‐limonene, accurately predicting these relative reactivities.

In summary, inverse vulcanization between sulfur and ENB is reported for the first time. The S‐ENB copolymers produced can stabilize a surprisingly high ratio of sulfur (up to 90 wt %) against depolymerization, in comparison with S‐DCPD (up to 80 wt %)^4^ and other inverse vulcanized polymers.^2^, ^11^The polymer is impervious to common solvents, there is no evidence of autoaccelaration as reported previously,^4^ and the polymer produces only a small volume of H_2_S in comparison with other crosslinkers (Supporting Information Table S6), which is beneficial to industrial scale up. The high sulfur ratios exhibited by S‐ENB will be crucial to many of the potential applications of sulfur polymers such as thermal and electrical insulation,^8^ LiS batteries,^6^, ^18^ and optical applications.^2^ The differences in properties of high‐sulfur polymers have been rationalized according to the reactive sites of their respective crosslinkers, with lower reactivity requiring higher polymerization temperature, thus causing increased hydrogen abstraction. These findings may make it easier to understand the differences in properties of other structurally diverse crosslinkers used to prepare inverse vulcanized sulfur‐polymers (e.g., farnesol, triglycerides, and renewable plant oils) 4, 18, 19, 20 as well as aiding in the selection of future potential crosslinkers and designing polymer blends.

## EXPERIMENTAL

### General Procedure for S‐ENB Synthesis

Sulfur (S_8_, masses shown in Supporting Information Table S1) was added to a 40 mL glass vial equipped with a magnetic stirrer bar and heated on a hot plate to 135 °C. Molten sulfur was formed (transparent, yellow solution) and to this, ENB (ENB masses shown in Supporting Information Table S1) was added drop wise via a Pasteur pipette. The mixture was heated at 135 °C for 20–30 minutes yielding a very viscous orange liquid. The product was then transferred to a mould and allowed to cure for ∼14 hours at 140 °C.

## Supporting information

Supporting Information
