# Supplementary material for: High sulfur content polymers: The effect of crosslinker structure on inverse vulcanization
Source: J Polym Sci A Polym Chem. 2018 Aug 24;56(16):1777–81. doi: 10.1002/pola.29067 (PMC6175008; doi:10.1002/pola.29067)
Supplement: Supplementary file 1 — Supporting Information [file POLA-56-1777-s001.pdf]

Supporting Information for

High sulfur content polymers: the effect of cross-linker structure on inverse vulcanisation

Jessica.A.Smith[a], Xiaofeng Wu[a], Neil. G. Berry[a] and Tom Hasell*[a]

a: Department of Chemistry, University of Liverpool, Crown Street, Liverpool, L69 7ZD

Materials: Sulfur (S_8, ≥99.5 %), Dicyclopentadiene (DCPD), 5 Ethylidene-2-norbornene >98.5 % purity, Chloroform-d ($CDCl_3$), Acetone, Acetonitrile, Toluene, THF, Methanol, and Hexane were purchased from Sigma Aldrich and used as received without further purification. 1,3-Diisopropenylbenzene (>97 %) was purchased from Tokyo Chemical Industry UK (TCI) and used as received.

Table S1: The sample compositions for the reactions carried out according to general procedure 3.3.

Reaction composition (wt. % ENB)	Mass of S_8 (g)	Mass of ENB (g)	Appearance
**10**	9.00	1.00	Rubber like black solid
**20**	8.00	2.00	Brittle black solid
**30**	7.00	3.00	Brittle Black solid
**40**	6.00	4.00	Brittle Black solid
**50**	5.00	5.00	Brittle black solid

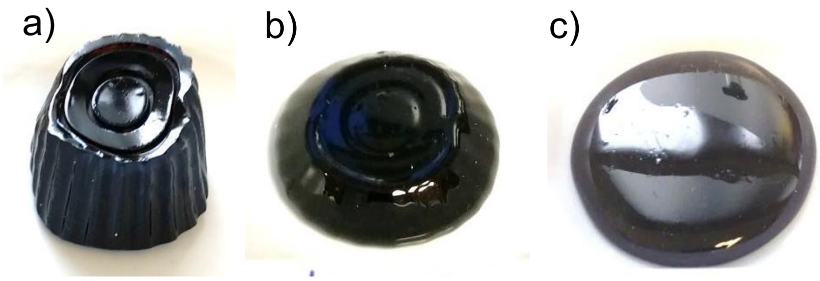

Figure S1. Photographs of a sample of sulfur-limonene polysulfide. (a) After removing from a mould b) After 2 hours at room temperature c) After 24 hours at room temperature.

Powder X-ray diffraction (PXRD): Powder X-Ray Diffraction (PXRD) patterns were carried out on samples using a PAN analytical X'pert powder diffractometer using CuKα radiation.

Differential Scanning calorimetry (DSC): Differential scanning calorimetry was carried out using Q2000 DSC (TA instruments). The method was a heat/cool/heat for three cycles; heating to 150 °C and cooling to – 80 °C at a heating rate of 5 °C/min with Tzero Hermetic pans.

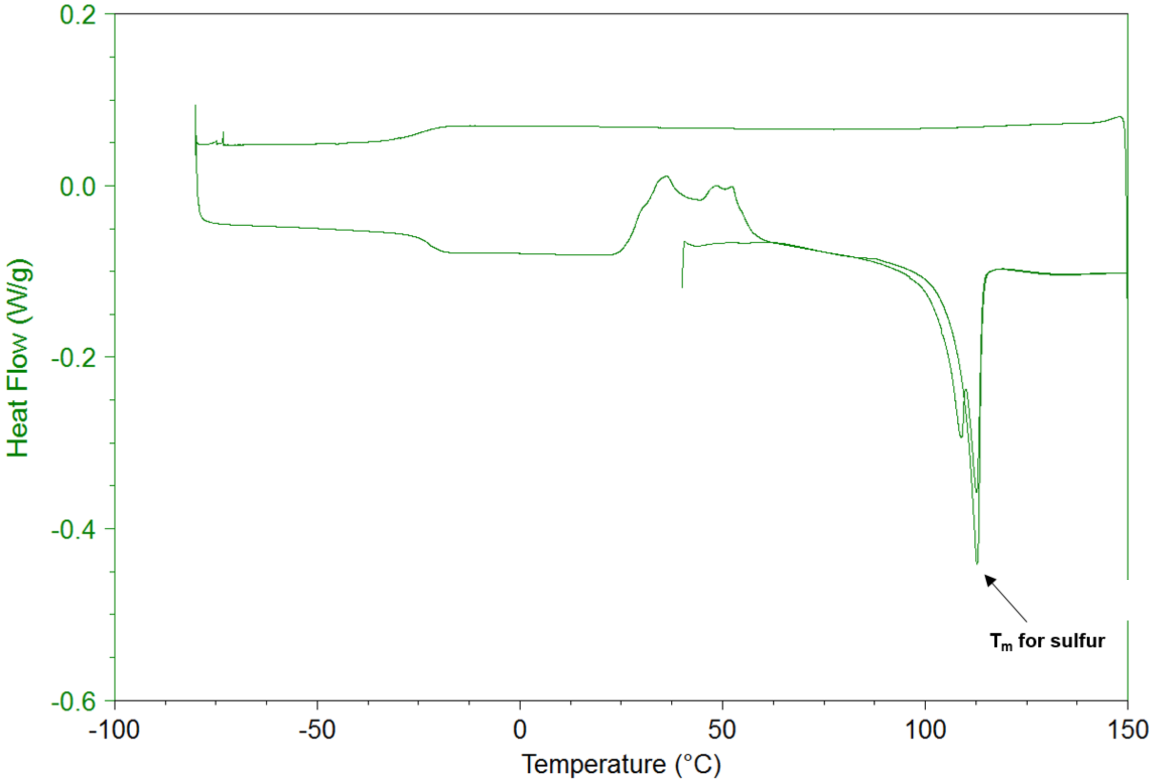

Figure S2. Representative DSC trace. Shown is 95 wt. % sulfur, 5 wt. % ENB co polymer, with a large T_m for elemental sulfur on both the first and second trace.

.

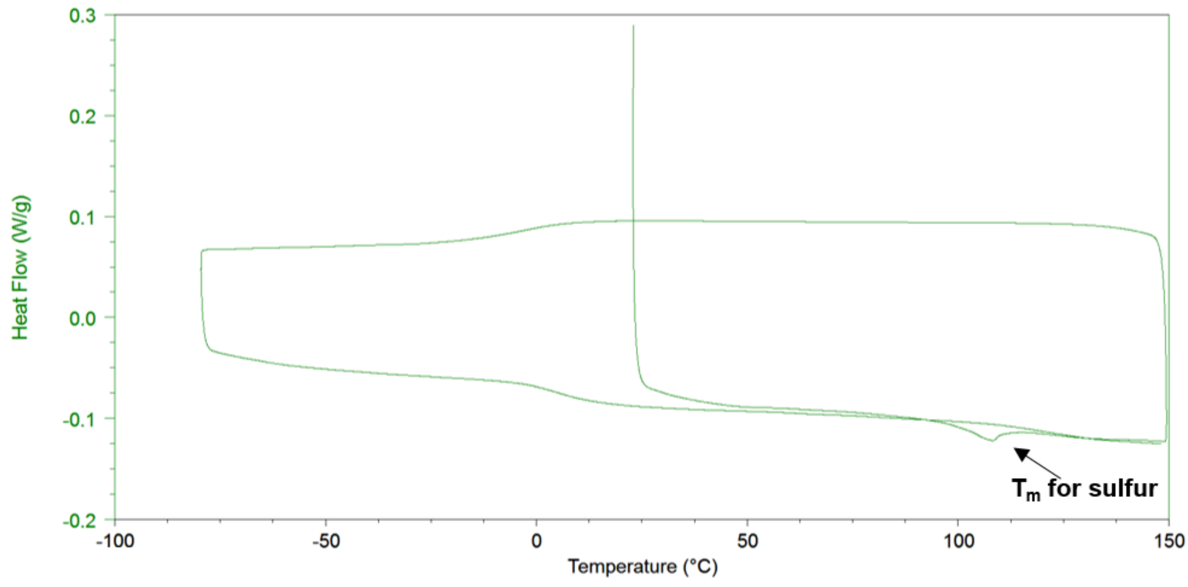

Figure S3. Representative DSC trace. Shown is 90 wt. % sulfur, 10 wt. % ENB copolymer, with a small T_m for elemental sulfur on the first trace. A slight peak at ~110 °C is observed on the first cycle only, and while this could correspond to the melting transition (T_m) of elemental sulfur, such transitions have also been recently attributed to crystallisation of a long chain polysulfides as present in inverse vulcanised polymers.[1,2]

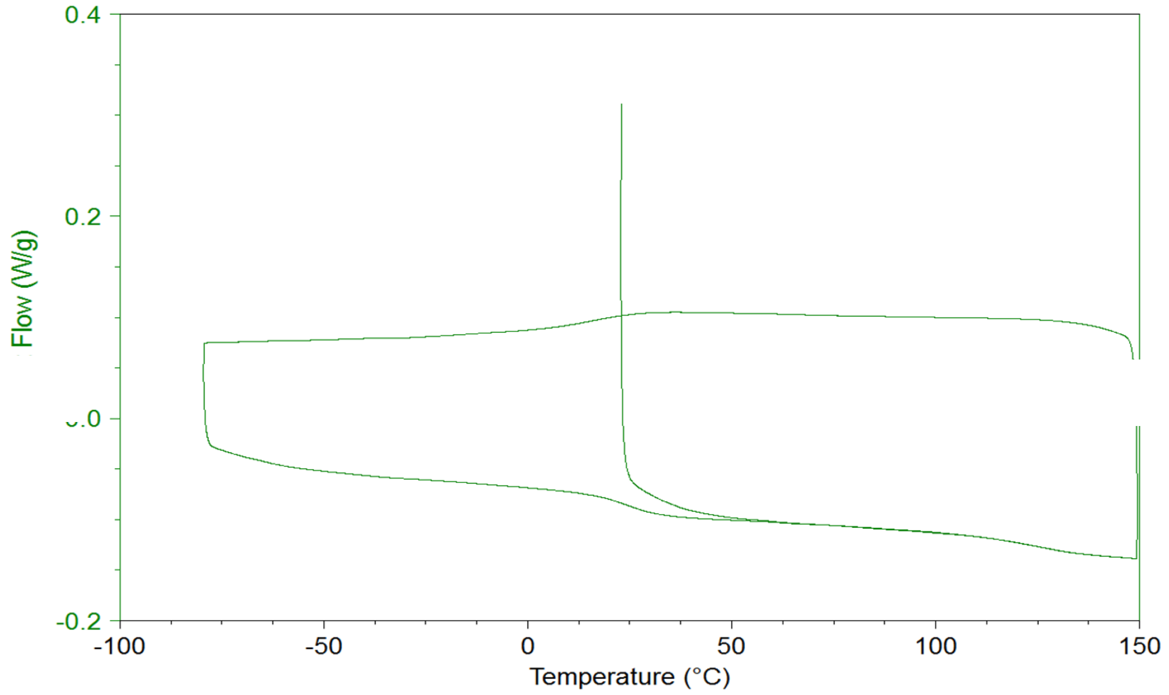

Figure S4. Representative DSC trace of 80 wt. % sulfur, 10 wt. % ENB copolymer, showing no melting transition for elemental sulfur.

Table S2: Elemental analysis of S-ENB polymers of 50, 60, 70 & 80 wt. % of elemental sulfur.

	**80:20 S-ENB**		**70:30 S-ENB**		**60:40 S-ENB**		**50:50 S-ENB**	
**Elements present**	Expected (%)	Actual (%)	Expected (%)	Actual (%)	Expected (%)	Actual (%)	Expected (%)	Actual (%)
**C**	17.99	16.44	26.98	24.49	35.92	30.76	44.97	39.42
**H**	2.01	1.60	3.02	2.57	4.02	2.00	5.03	4.23
**S**	80.00	82.29	70.00	73.28	60.0	65.60	50.00	56.91

Thermal gravitational analysis (TGA): TGA was carried out using platinum pans, samples were heated to 900 °C under a N_2 atmosphere and then held for 10 minutes under air to burn any remaining material.

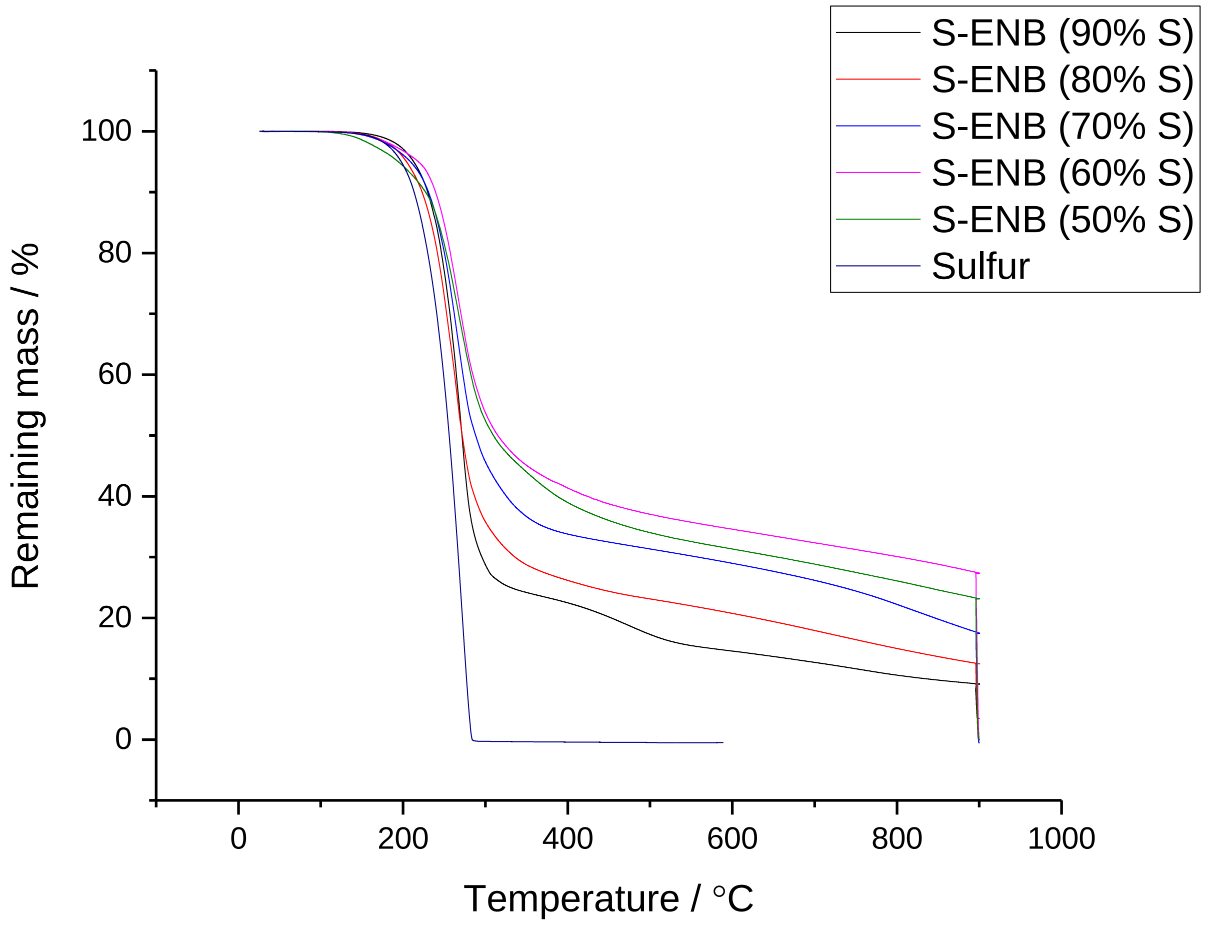

Figure S5. TGA thermograms of poly(S-ENB) materials with varying compositions of ENB and sulfur. ENB was too volatile to obtain TGA data, however there is a clear decomposition for sulfur at ~ 250 °C and an increasing percentage of char mass remaining as the composition of ENB increases, suggesting a copolymer has successfully been synthesised.

FTIR: Fourier transform infrared spectroscopy was carried out using Thermo Nicolet IR 2000 FT-IR using the KBr method.

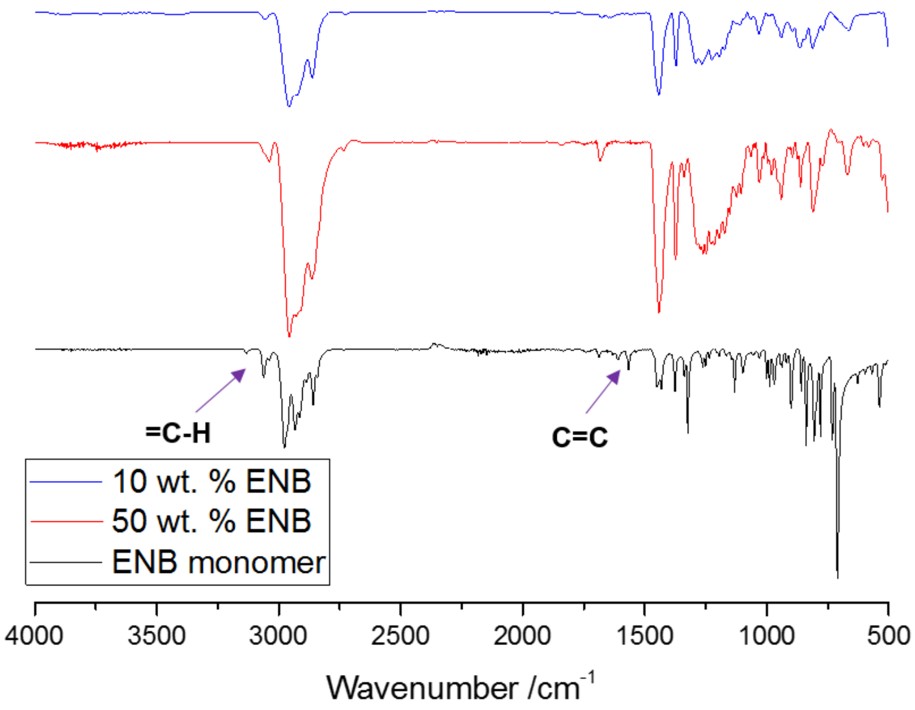

Figure S6. FTIR spectra of ENB and S-ENB copolymers containing 10 wt. % and 50 wt. % ENB.

Solubility studies: S-ENB (10 mg) of compositions 10- 50 wt. % of ENB were added to a 12 mL vial. To this 10 mL of different solvents were added (acetone, acetonitrile, chloroform,

hexane, methanol, THF, toluene, water). The solutions were left stirring for ~24 hours on a tube roller.

Table S3: Results were determined by >100 mg solid in 10 mL solvent and stirring overnight. Solubility's are given in mg/mL, IS= insoluble, trace= colour visible in solution but <1 mg/ mL.

	Crosslinker								
Solvent	*	DIB	Limonene	Myrcene	Farnesene	Farnesol	DCPD	ENB (50 wt. %)	ENB (20 wt. %)
	Acetone	1.6	trace	IS	trace	trace	IS	IS	IS
	Acetonitrile	10.9	trace	IS	IS	IS	IS	IS	IS
	Chloroform	9.7	>17.2	9.7	20.8	25.7	IS	IS	IS
	Hexane	IS	trace	trace	trace	trace	IS	IS	IS
	Methanol	IS	trace	IS	IS	IS	IS	IS	IS
	THF	10.5	>12.4	11.7	16.8	25.1	IS	<1	IS
	Toluene	13.6	>16.1	12	19.6	13	IS	IS	IS
	Water	IS	IS	IS	IS	IS	IS	IS	IS

NMR kinetics: A reaction of 50:50 S_8: ENB was prepared, however was not cured overnight. Two separate experiments were carried out heating the reaction mixture at 135 °C and 160 °C.

~20 mL aliquots of the reaction mixture were taken from the vial and dissolved in ~10 mL of deuterated chloroform. An aliquot of the reaction mixture was taken at 15, 30, 60 and 75 minutes for each experiment.

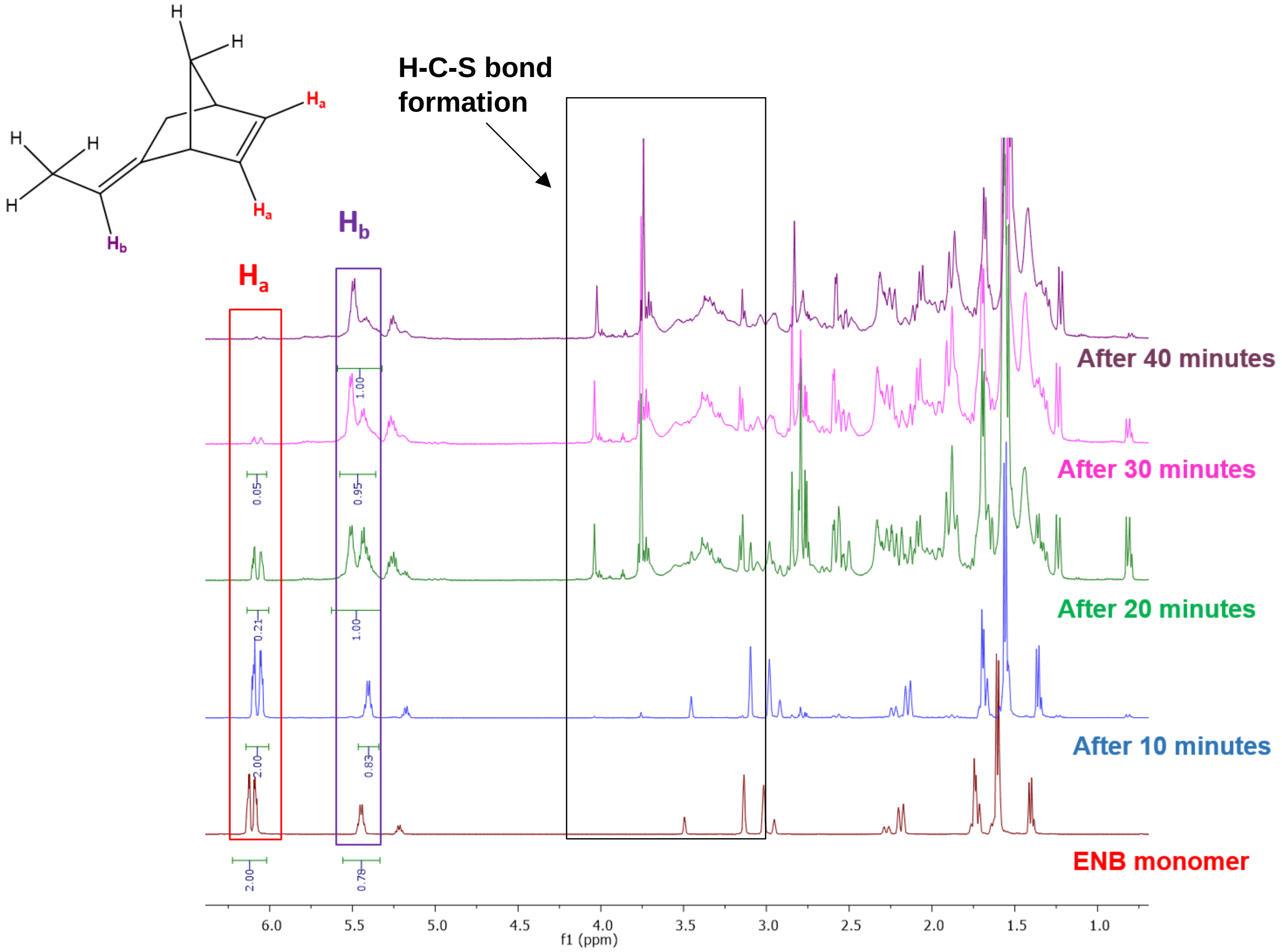

Figure S7: NMR kinetics experiment conducted at 160 °C. ~20 mL aliquots were dissolved in $CDCl_3$ and were taken at 10, 20, 30 and 40 minutes.

Table S4: Ratios of Ha: Hb at different times of the NMR kinetics experiment conducted at 160 °C.

Time (minutes)	Ratio of Ha:Hb
10	2:0.83
20	0.21:1
30	0.05:0.95
40	0:1

Table S5: Ratios of Ha: Hb at different times of the NMR kinetics experiment conducted at 135 °C.

Time (minutes)	Ratio of Ha:Hb
15	2:1
30	0.74:1
60	0.06:1
75	0.06:1

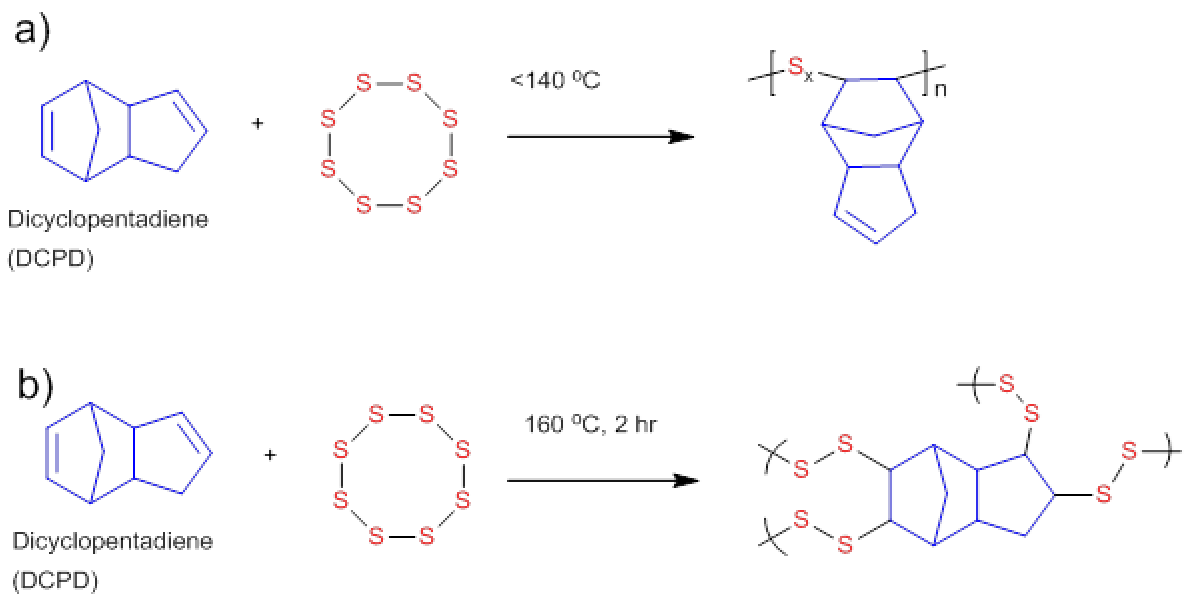

Figure S10: Different potential reactions of sulfur with DCPD leading to linear (a) or network (b) polymers.

Calculating Fukui indicies:

Spartan '16 V.2.0.3 (https://www.wavefun.com/) software was used to model crosslinkers and calculate Fukui indices. Limonene, DIB, ENB and DCPD were built and initially energy minimised using a MMFF94 forcefield. Equilibrium geometry calculations at ground state in gas with density functional theory (ωB97X-D, 6-31G*, total charge = neutral, unpaired electrons = 0). Single point energy energy calculations were then performed after this at ground state in gas with the same functional and basis set in both the anionic and cationic states (unpaired electron = 1). Natural atomic charges were used to calculate the condensed Fukui indices using the equation below.

Fukui function for radical attack = $(q_{N-1}^{A} - q_{N+1}^{A})/2$

Where q_{N+1}^{A} and q_{N-1}^{A} are partial charges of atom A in the molecule with N+1 electrons and N-1 electrons, respectively.

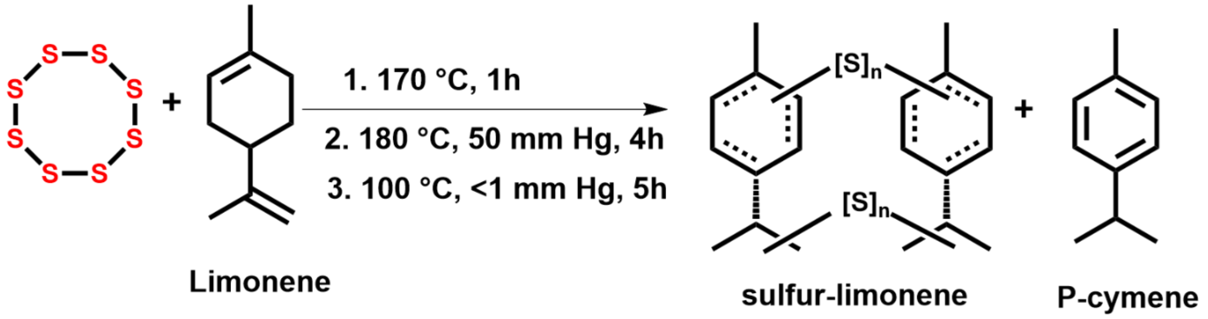

Figure S11: Reaction between sulfur in limonene as reported by Chalker *et al.*[3] p-cymene was detected as a by-product. However, even after removal of this under extensive high vacuum, there remain aromatic signals present in the NMR of the polymer produced, which is soluble.

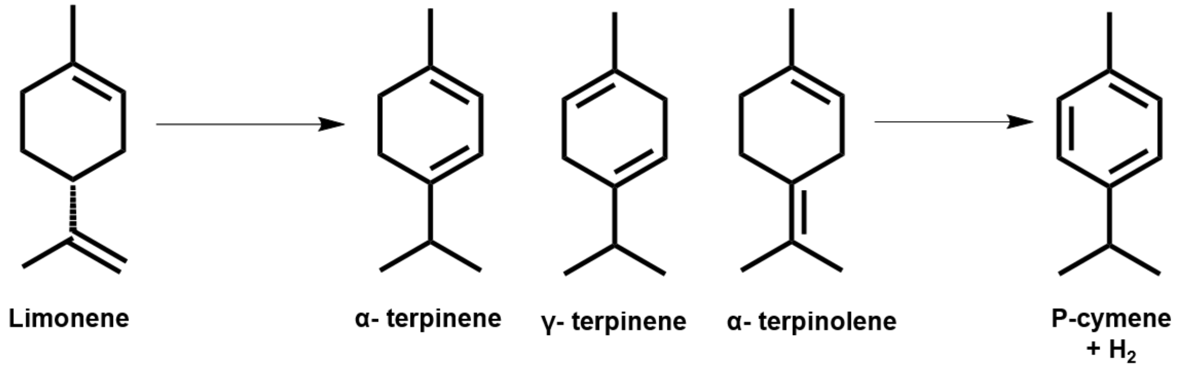

Figure S12: Limonene is subject to 1-3 hydrogen shifts, and loss of hydrogen to form *p*-cymene,[4] especially when reacted with sulfur at high temperatures.[5]

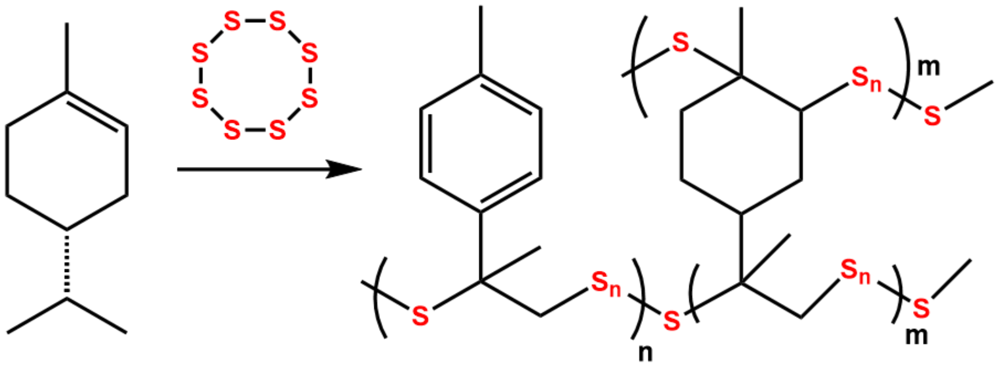

Figure S13: Scheme showing a representation of the hypothesized structure of sulfur-limonene, containing both linear (a), and branching (b) moieties. Loss of hydrogen from limonene is known to produce 1-methyl-4-(2-propenyl)benzene, p,α-dimethyl styrene (DMS).[6] This must proceed with loss of hydrogen. This presumably occurs in the form of H_2S, which we have detected produced from the reaction in significant quantities. Formation of DMS will lead to linear polymer subunits (a). Reaction of un-dehydrogenated limonene will lead to branching units (b). The relative solubility any low T_g suggests (a) to be the major component.

Gas capture:

Sulfur (5 g) and crosslinker (5 g, Table 5) were added to a 40 mL reaction vial with a stirrer bar. The vial was sealed with a rubber septum and connected with a tube ended with a needle to a measuring cylinder (100 mL). The measuring cylinder was pre filled with deionised water and was placed upside down in a 1 L beaker with water. The reaction mixture was heated to the normal reaction conditions for each crosslinker (Table S6) until each reaction produced no more gas.

Table S6: The volume of gas collected for the reaction between sulfur with Limonene, DCPD and ENB at different reaction temperatures.

Crosslinker	Temperature of reaction (°C)	Volume of gas collected (mL)	Maximum sulfur stabilisation reported (wt. %)
Limonene	180	63.0	50
DCPD	165	26.5	80
ENB	135	9.5	90

The largest volume of gas was collected for the reaction between sulfur and limonene (63 mL), the gas is thought to be mainly H_2S and did trigger a H_2S detector (alarm limit 2.4 ppm) on exposure. However, the H_2S detector was not triggered by the gas produced in the reactions between sulfur and ENB. The gas released by the reaction of ENB with sulfur may mostly be ambient gasses dissolved in the monomers at room temperature, and released on heating, with H_2S release negligible at these reaction conditions.

The higher the reaction temperature the larger the volume of gas collected. Each reaction was therefore conducted again at 135 °C to see the effects of reaction temperature (Table S7). The

reaction for sulfur and ENB cannot be conducted any higher than 135 °C due to the boiling point of ENB being 147.6 °C.

Table S7: The volume of gas collected for the reaction between sulfur with Limonene, DCPD and ENB at 135 °C for 1 hour.

**Crosslinker**	**Temperature of reaction (°C)**	**Volume of gas collected (mL)**
Limonene	135	<1 – no reaction
DCPD	135	~5 – only very slight reaction
ENB	135	9.5 – full reaction

However, reactions of sulfur with both limonene and DCPD at 135 °C results in an incomplete reaction with the gases collected being negligible. The volume of gas collected is most likely the effects of both temperature and how susceptible the crosslinker is to losing hydrogen. It is important to note that there is a clear relationship between the volume of gas collected and the wt. % of sulfur that each crosslinker stabilises Table S6.

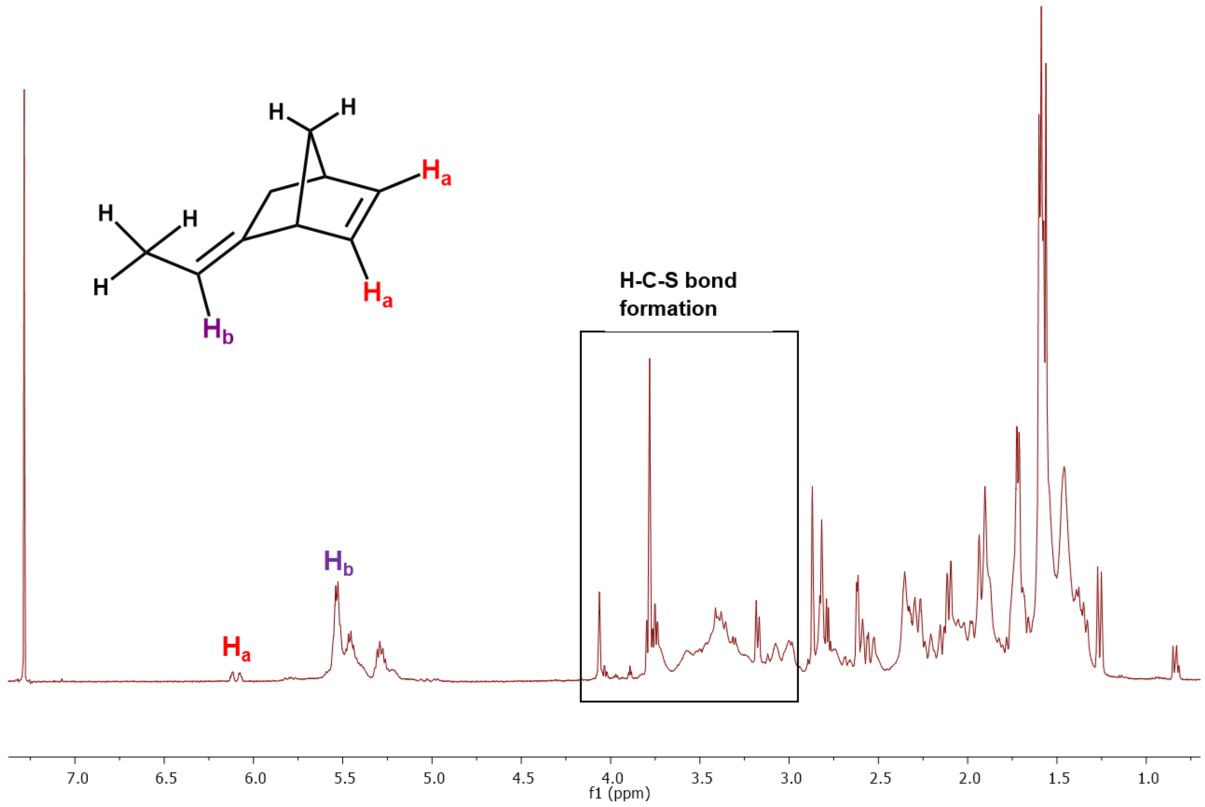

Figure S14: NMR of reaction between 50 wt. % sulfur, 50 wt. % ENB after 40 minutes. NMR shows no evidence of aromatic protons. Reaction was conducted at 160 °C.

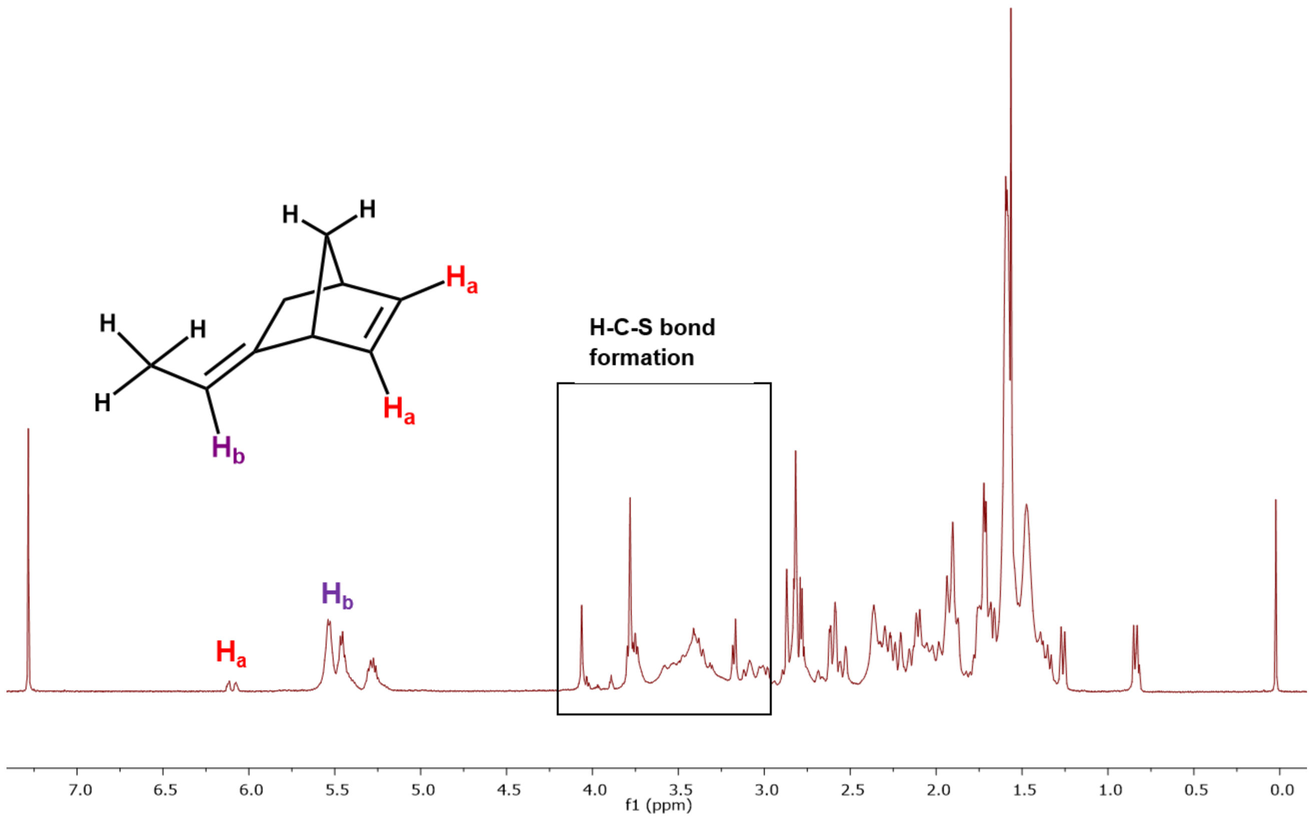

Figure S15: NMR of reaction between 50 wt. % sulfur, 50 wt. % ENB after 75 minutes. NMR shows no evidence of aromatic protons. Reaction was conducted at 135 °C.

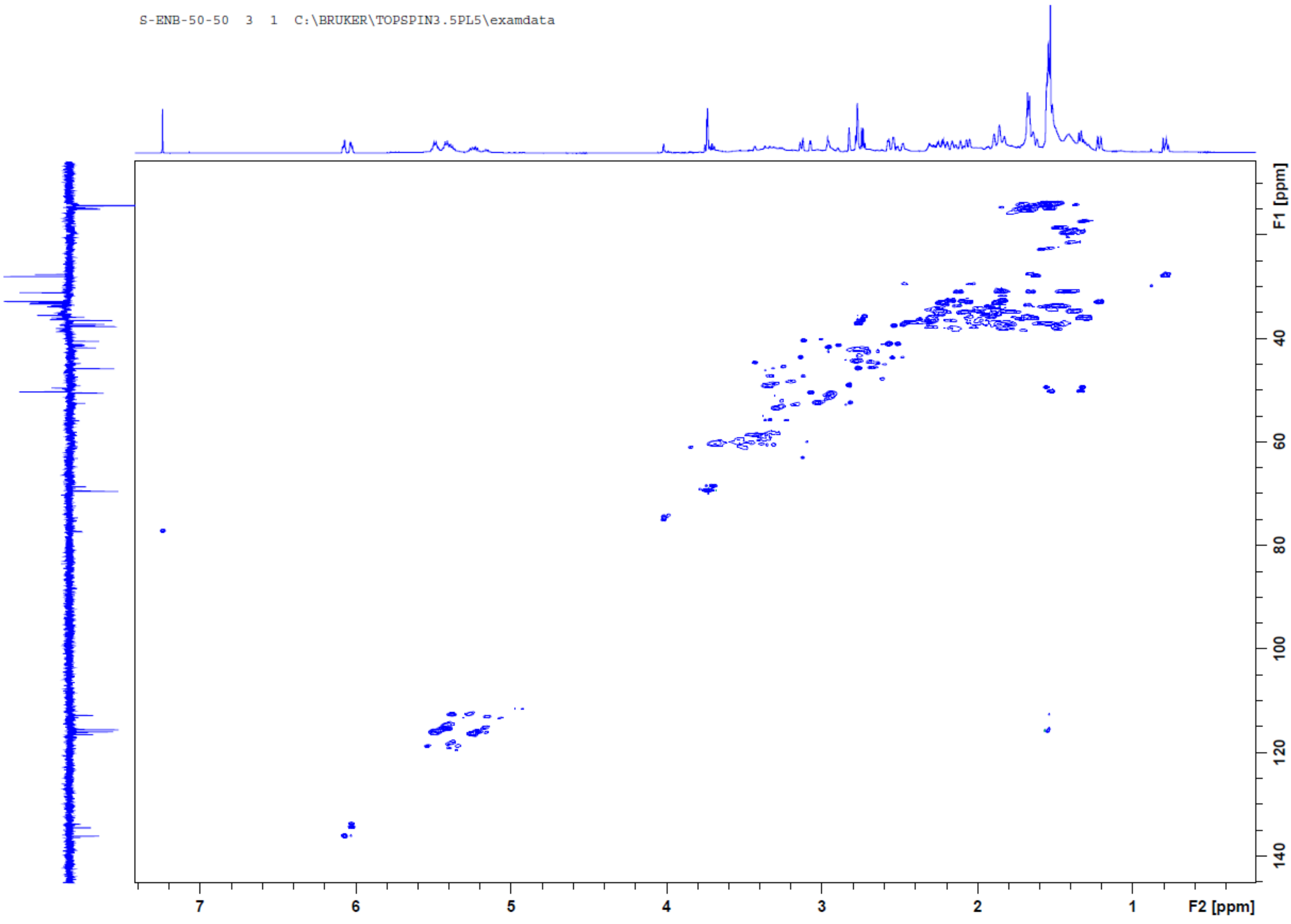

Figure S16: HSQC of 50 wt. % sulfur, 50 wt. % ENB reaction after 60 minutes at 135 °C. There is no evidence of any aromatic by product forming at this reaction time.

References

1 W. J. Chung, J. J. Griebel, E. T. Kim, H. Yoon, A. G. Simmonds, H. J. Ji, P. T. Dirlam, R. S. Glass, J. J. Wie, N. a Nguyen, B. W. Guralnick, J. Park, A. Somogyi, P. Theato, M. E. Mackay, Y. Sung, K. Char, J. Pyun, Á. Somogyi, P. Theato, M. E. Mackay, Y. Sung, K. Char, J. Pyun, A. Somogyi, P. Theato, M. E. Mackay, Y. Sung, K. Char, J. Pyun, Á. Somogyi, P. Theato, M. E. Mackay, Y. Sung, K. Char and J. Pyun, *Nat. Chem.*, 2013, **5**, 518–524.

2 A. Hoefling, Y. J. Lee and P. Theato, **201600303**, 18–21.

3 M. P. Crockett, A. M. Evans, M. J. H. Worthington, I. S. Albuquerque, A. D. Slattery, C. T. Gibson, J. A. Campbell, D. A. Lewis, G. J. L. Bernardes and J. M. Chalker, *Angew. Chemie - Int. Ed.*, 2016, **55**, 1714–1718.

4 M. A. Martin-Luengo, M. Yates, E. S. Rojo, D. Huerta Arribas, D. Aguilar and E. Ruiz Hitzky, *Appl. Catal. A Gen.*, 2010, **387**, 141–146.

5 O. Illa, M. Namutebi, C. Saha, M. Ostovar, C. C. Chen, M. F. Haddow, S. Nocquet-Thibault, M. Lusi, E. M. McGarrigle and V. K. Aggarwal, *J. Am. Chem. Soc.*, 2013, **135**, 11951–11966.

6 S. A. Sanchez-Vazquez, T. D. Sheppard, J. R. G. Evans and H. C. Hailes, *RSC Adv.*, 2014, **4**, 61652–61655.
